# Gothenburg very early supported discharge: evaluating differences in costs and environmental impact due to rehabilitation consumption during the first year in patients hospitalized due to mild stroke

**DOI:** 10.1186/s12913-025-12509-y

**Published:** 2025-03-19

**Authors:** Lena Rafsten, Annie Palstam, Katharina S. Sunnerhagen

**Affiliations:** 1 Institute of Neuroscience and Physiology, Dept of Clinical Neuroscience and Rehabilitation Medicine, Sahlgrenska Academy, University of Gothenburg, Gothenburg, Sweden; 2 Department of Occupational Therapy and Physiotherapy, Sahlgrenska University Hospital, Gothenburg, Sweden; 3 Department of Neurology, Sahlgrenska University Hospital, Gothenburg, Sweden

**Keywords:** Stroke, Early supported discharge, Sustainability, Rehabilitation, Economy

## Abstract

**Background:**

It has been showed that Early Supported Discharge could decrease the length of hospital stay and thus a cost saving alternative to conventional in-hospital stroke rehabilitation. This might lead to decreased environmental impact due to reduced need of in-hospital care but has not been evaluated.

**Methods:**

One hundred forty adult patients from a stroke unit at the Sahlgrenska University Hospital who were consecutively included in the GOTVED study and then randomized to Very Early Supported Discharge (VESD) group or to a control group who received ordinary discharge was included. Descriptive data are presented as mean ± standard deviation (SD) or median and interquartile range (IQR), as appropriate. The chi-square test or Mann-Whitney U test was used to test for group difference. A two-sided value of *p* ≤ 0.05 was considered to represent statistical significance.

**Results:**

The VESD group had an average hospital stay that was 2 days shorter than that of the control group, resulting in a mean cost of € 11,151 in the VESD group, and for the control group € 10,741 (*p* = 68). The mean environmental impact in kg CO_2_ emissions was 525.3 (± 240.1) kg CO_2_/patient in the VESD group, VESD visits included, and 552.5 (± 284.2) kg CO_2_/patient in the control group (*p* = 0.55).

**Discussion:**

Despite the fact that the 2-day difference in hospital stay between the VESD group and the control group was not statistically, if generalized a two days shorter in-hospital stay to all hospitalized stroke patients in Sweden, would results in a saving of € 6,944,500 /year. Two days shorter in-hospital days would also mean a reduction in CO_2_ emissions with approximately 360,050 CO_2_ kg /year. These hypothetical possible reductions in healthcare emissions would contribute to more sustainable healthcare.

**Conclusion:**

A policy of offering more patients VESD after stroke could reduce healthcare costs and environmental impact, contributing to sustainable healthcare.

**Trial registration:**

clinicaltrials.gov: NCT01622205, 2012-06-18.

## Introduction

In Sweden, around 25,000 people suffer a stroke each year, of which 22,000 have their first stroke. Of these 25,000, 5600 patients die during the first year and close to 10,000 of the patients suffer a stroke that leaves them with life-long disabilities [1]. The cost of one stroke in Sweden during 2016 € (euro) 37,000‒50,000 depending on stroke type and if it is the first or recurrent stroke incidence [2]. For example, the inpatient days the first year costs € 740–867 per day depending on stroke type, and an outpatient visits to primary care costs € 287 per visit [2]. This gives a yearly total healthcare cost of € 10.7 million for healthcare consumption related to all first stroke cases in Sweden.

However, the costs of healthcare activities are not only monetary but include a substantial environmental impact, since healthcare activities globally contribute approximately 5% of total greenhouse gas emission, corresponding to the emissions produced by the largest countries in the world [3]. Therefore, sustainability ought to be a domain in evaluation of healthcare, taking economic, social and environmental aspects of sustainability into consideration while providing healthcare to meet the needs of present patients and population without compromising the ability to meet future needs [4].

The Sahlgrenska University Hospital is the largest hospital network in Sweden, providing emergency and basic care to 700,000 inhabitants in the Gothenburg region and highly specialized care to 1.7 million inhabitants in the region. Locally, healthcare activities in Region Västra Götaland, a county in western Sweden, account for 21% of total greenhouse gas emissions, equal to just over 3 million tons of carbon dioxide (CO_2_) equivalents per year, or around 3% of the total consumption-based emissions in Sweden [5].

Financial costs are sometimes anticipated as a major drawback of the transition to sustainable healthcare [4]. On the contrary, a more sustainable healthcare system will increase the likelihood of preventing illness and improving efficiency in healthcare, meaning that financial savings can be made at the same time as environment impact will decrease [6]. Leading the way towards sustainable healthcare, the National Health Service in England has taken a national initiative to map greenhouse gas emissions from healthcare and has set a net zero target for emissions by 2040, for the sake of present and future public health [7].

In an era where insufficient resources mean difficult prioritizations in healthcare, when rehabilitation is often down prioritized in relation to more acute healthcare activities, it is of great interest to investigate the consumption and cost of rehabilitation after stroke. A systematic review from 2021 concluded that the economic burden of stroke has increased and that Sweden has the second highest average cost per stroke patient per year in the world with a cost of 44,144 € [8]. This may be since stroke care in Sweden is well-developed, including stroke cameras in the ambulance, an AI (artificial intelligence) app that helps with the interpretation of radiological images, and high proportion of patients receiving thrombolysis and thrombectomy within 60 min. In Sweden, there are approximately 80 hospitals and 90% (72) of them have a stroke unit. In 2022, 93% of the stroke patients in Sweden were cared for in a stroke unit [9].

Early supported discharge (ESD) with continued rehabilitation in the home from a multidisciplinary stroke team has been shown to be beneficial [10]. ESD can also decrease the length of hospital stay [11] and be a cost-saving alternative to conventional in-hospital stroke rehabilitation [12]. This is in line with studies concluding that a shift from a centre-based to a home-based approach to stroke rehabilitation can provide good value for money in most European countries [13]. The reduced need for in-hospital care should decrease its environmental impact, although this has not been evaluated. In our previous study Gothenburg Very Early Supported Discharge (GOTVED) we used the term very early supported discharge (VESD) due to the shortened hospital stay in Sweden the last years [14]. In the GOTVED study, we evaluated patient outcomes over time, but the economic costs and environmental impact of rehabilitations over time have not yet been investigated.

We therefore aimed to evaluate the cost and environmental impact of rehabilitation consumption during the first year after discharge, in patients who were randomized to very early supported discharge (VESD) compared with patients who were discharged from hospital according to ordinary routines after stroke.

## Methods

**Fig. 1:**
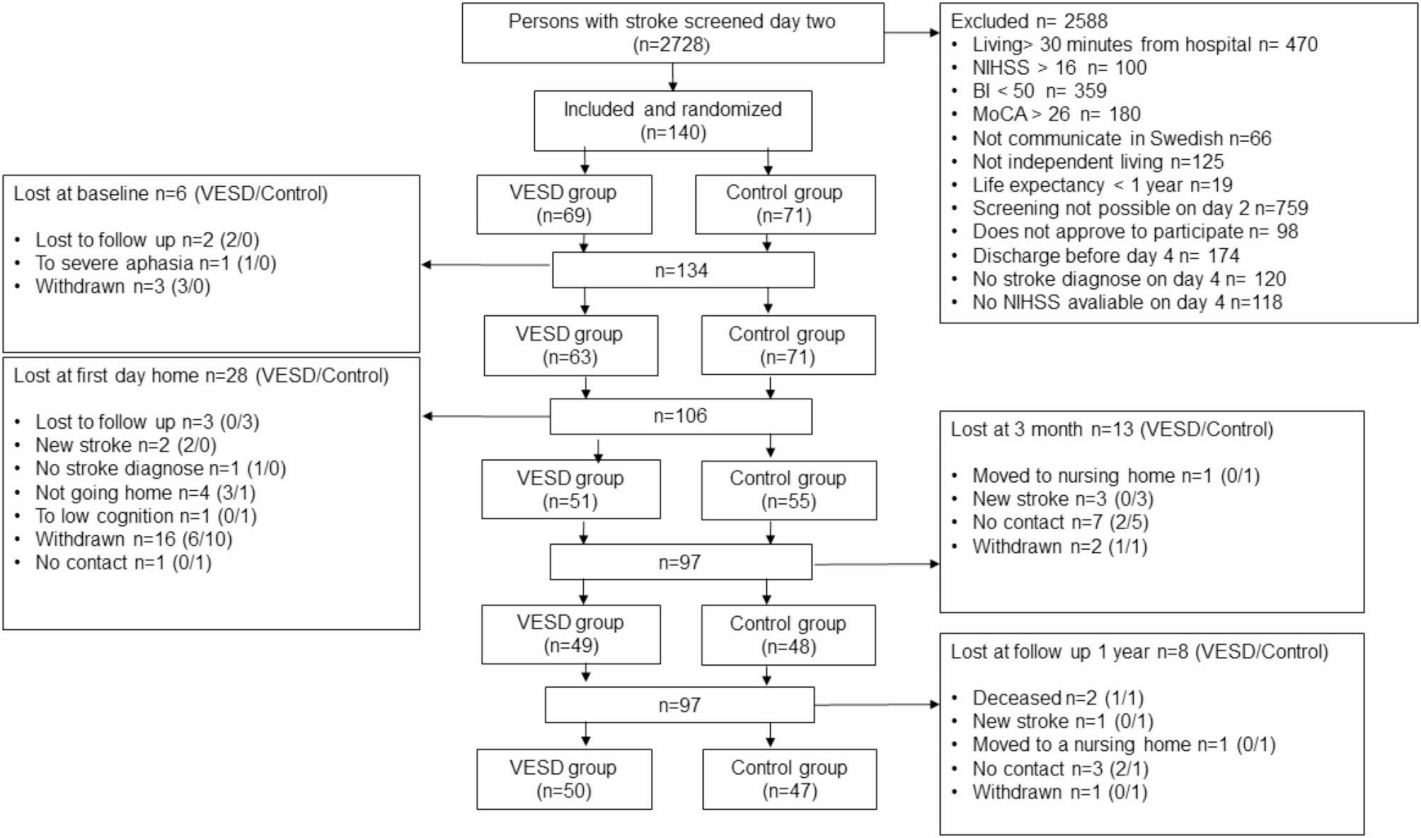
Flow chart of patients allocated to the GOTVED study. BI: Barthel Index, HADS-A: Hospital Anxiety and Depression Scale-Anxiety subscale, MoCA: Montreal Cognitive Assessment, mRS: modified Rankin Scale, NIHSS: National Institute of Health Stroke Scale, VESD: very early supported discharge

This study is a secondary analysis of data from a randomized controlled study, Gothenburg Very Early Supported Discharge (GOTVED); clinicaltrials.gov: *NCT01622205* [14]. Therefore not powered for this analysis. Over a period of 5 years, from September 2011 to April 2016, 140 adult patients from a stroke unit at the Sahlgrenska University Hospital were consecutively included in the study and then randomized to either a group who received VESD or the control group who received ordinary discharge (Fig. 1).

Data for patients treated in Sahlgrenska University Hospital and included in the GOTVED study were retrieved from the study protocol and from patient medical records. Data from outpatient rehabilitation from community and primary care, and data from domestic service, community care and special transport service were retrieved from the Västra Götaland regional registry (VEGA registry). Regarding interventions, the following ICD-10 codes for main diagnoses were used: I61, I62, I63, I64, I67 and I69. Readmission within 3 days was counted as one inpatient session. The VESD team is organized as a part of the stroke unit at the hospital; intervention by the team has therefore been included in the cost for the inpatient care.

The term rehabilitation consumption was used to include all rehabilitation visits to outpatient hospital and primary care such as clinical examinations and rehabilitation post discharge from the hospital. Since patients were included between 2011 and 2016 and the data were retrieved in 2019, the sums received were transformed from SEK to € at the exchange rate for April 2019 (exchange rate: € 1 = SEK 10.5) [15]. A set sum per care level was given by the VEGA registry as follows: inpatient care, 746.4 €/day; VESD visit, 105.7 €/visit; and primary care rehabilitation, 54.8 €/visit.

In this study the impact of inpatient care and rehabilitation on the environment has been estimated in kg CO_2_, according to estimates from the National Health Services in England [7]. A bed-day at the stroke unit has been estimated as a “Low intensity inpatient bed-day” which according to the Sustainable Development Unit (SDU) corresponds to an estimated 37.9 kg CO_2_/day [7]. A home visit from the VESD team has been estimated to emit 3.7 kg CO_2_/visit. This value assumes that each VESD visit involves a 20 km round trip and that a trip of one English mile implies emission of 0.3 kg CO2 according to the Sustainable Development Unit (SDU) [7]. A visit to the primary care rehabilitation has been estimated to emit 9.9 kg CO_2_.

### Statistical methods

Descriptive data are presented as mean ± standard deviation (SD) or median and interquartile range (IQR), as appropriate. The chi-square test or Mann-Whitney U test was used to test for group difference. A two-sided value of *p* ≤ 0.05 was considered to represent statistical significance.

## Results

Of 140 patients included in the GOTVED study, 42 dropped out of the study during the first year after stroke incidence: 20 in the VESD group and 22 in the control group. Since we performed intention to treat analyses, their data were included in the study. The control group stayed on average 2 days longer in hospital compared with the VESD group although it was not statistically significant (Table 1). The mean cost of the inpatient care in the VESD group was 886.6 (± 356.6) €/day, in total 11,151(± 5780.9) €, the VESD visits included, and for the control group 741 (± 270.7) €/Day, in total 10,741(± 6123.6) € (*p* = 0.46). The VESD group consumed a mean of 10.2 (± 4.9) visits from the VESD team during 4 weeks (Table 1).

**Table 1:** Characteristics of included patients

Characteristics	All patients	VESD group (*n* = 69)	Control group (*n* = 71)	*p*-value
**Age**				
Mean (SD)		75.5 (11.1)	72.7 (12.4)	*0.17*
Median (IQR)		78 (69.5–84)	75 (65–82)	
Male (%)		42 (60.9)	44 (62)	*0.89*
**Hospital days** (n)				
Mean (SD)	13.9 (7.1)	13.1 (6.6)	14.7 (7.3)	*0.18*
Median (IQR)	12 (9–17)	11 (9–17)	13 (9–18)	
**Thrombolysis % (n)**	12 (8.6)	6 (8.7)	9 (12.7)	*0.58*
**Thrombectomy % (n)**	6 (4.3)	3(4.3)	3 (4.2)	*1.0*
**NIHSS**^a^ (*n* = 137)	1 (0–3)	1 (0–3)	1 (0–4)	*0.28*
**BI**^a^ (*n* = 139)	80 (65–90)	80 (70–90)	80 (60–90)	*0.37*
**Hospital cost (2019 €)**	1 440 541,7	677 910,3	762 631,4	*0.34*
Mean (SD)	10,289.6 (5848.1)	9824.8 (5556.4)	10,741.3 (6123.6)	*0.34*
Median (IQR)	8449.6 (5859.4–12787.3)	8160.8 (5648.3–12044.2)	8816.7 (5912.1–14186.6)	
**Total cost hospital + VESD (2019 €)**				
Mean (SD)		11,151.1 (5780.9)	10,741.3 (6123.6)	*0.46*
Median (IQR)		9543.3 (6823.7–13,426.7)	8816.7 (5912.1–14186.6)	
**VESD visits total** (n)		551		
**VESD visits /patient** (n)				
Mean (SD)		10.2 (4.9)		
Median (IQR)		11 (7–14)		
**VESD cost (2019 €)**		60 169.2		
Mean (SD)		1122.9 (609.5)		
Median (IQR)		1172 (746–1492)		
**EQ5D (VAS 0–100)** (*n* = 92)				
1 year after stroke incidence				
Mean (SD)	76.1 (21.9)	76.3 (21.9)	75.9 (22.2)	*0.87*
Median (IQR)	80 (64–90)	84 (64–95)	80 (63.3–90)	

*BI* Barthel Index, *Eq. 5D* EuroQoL 5 dimensions, *€* Euros, *IQR* inter quartil range, *NIHSS* National Institute of Health Stroke Care, *SD* standard deviation, *VESD* very early supported discharge

^a^Second day (36–48 h) after arrival at the stroke unit

### Rehabilitation consumption and cost post discharge from hospital

Of the 140 patients included in the study 82 (58.6%) used primary care rehabilitation: 37 (53.6%) in the VEDS group and 45 (63.3%) in the control group. The VESD group (4 weeks intervention) consumed on average 6.7 (± 10.8) primary care rehabilitation visits per patient compared with 7.7 (± 13.4) in the control group (*p* = 0.72) (Table 2). The VESD group consumed significant more visits from the occupational therapist, 86 visits, than the control group, 69 visits (*p* = 0.04). The mean sum of cost for outpatient rehabilitation in the VESD group was 432.9 (± 753.6) € compared with 505.7 (± 924.1) € in the control group (*p* = 0.86), with a total sum of costs for outpatient rehabilitation of 63,498.3 € (Table 2).

**Table 2:** Cost and consumption of primary care rehabilitation during first year post discharge

Characteristics	All Patients (*n* = 140)	VESD group (*n* = 69)	Control group (*n* = 71)	*p*-value
**Number of patients who consumed primary care rehabilitation**	82	37	45	
**Number of visits**	1004	456	548	*0.36*
**Number of visits/patient**				
Mean (SD)	7.2(12.2)	6.7 (10.8)	7.7 (13.4)	*0.72*
Median (IQR)	1 (0–8)	1 (0–9)	1 (0–8)	
**Different professionals regarding all visits with a registered Stroke diagnose**				
Physiotherapist	753	325	428	*0.57*
Occupational therapist	155	86	69	0.04*
Speech therapist	93	44	49	*0.51*
Unidentified primary rehabilitation	3	1	2	
**Total cost (2019 €)**	63,498.3	27,924.4	35,573.8	
Mean (SD)	470.9 (844)	432.9 (753.6)	505.7 (924.1)	*0.86*
Median (IQR)	82.8 (0–635.3)	55.3 (0–607.6)	110.4 (0–662.8)	

*IQR* inter quartile range, *€* Euros, *SD* standard deviation

* *p*<0.05

### Environmental impact

In the regard to the environmental impact of care in kg CO_2_ emissions, the inpatient care accounted for a mean of 525.3 (± 251.3) kg CO_2_/patient in the VESD group, the VEDS visits included, and 552.5 (± 284.2) kg CO_2_/patient in the control group, a difference of 27.2 kg CO_2_/patient in favour of the VESD group (*p* = 0.85). The mean environmental impact from primary care rehabilitation visits was 9.9 (± 106.9) kg CO_2_/patient in the VESD group and 9.9 (± 132.9) kg CO_2_/patient in the control group (*p* = 0.72) (Table 3).

**Table 3:** Environment burden in kg carbon dioxide (CO_2_)/ mean /patient

Characteristics	All patients (*n* = 140)	VESD group (*n* = 69)	Control group (*n* = 71)	*p*
Inpatient care mean CO_2_/patient (SD)	539.1 (267.8)	495.7 (252.3)	552.5 (284.2)	*0.23*
VESD visits mean CO_2_/patient (SD)		29.5 (22.6)		
Total inpatient mean CO_2_/patient (SD)		525.3 (251.3)	552.5 (284.2)	*0.85*
Primary care rehabilitation mean CO_2_/patient (SD)	121.6 (136.8)	9.9 (106.9)	9.9 (132.9)	*0.72*

*CO*_2_ carbon dioxide, *SD* standard deviation, *VESD* very early supported discharge

## Discussion

There was a 2-day difference in hospital stay between the VESD group and the control group with the control group staying a median of 2 days longer, however not statistically different. The mean cost of a hospital stay was 10,289 €/patient; with a mean stay of 13.9 days in hospital, this was 731 €/day. It could be argued that these are fairly small amounts of money and emissions and therefore of limited importance, but with approximately 25,000 people suffering from stroke each year in Sweden, whereof about 19% receive VESD [9], extrapolations of costs and emissions saved indicate potentially large benefits. Assuming that the 2-days shorter in-hospital stay is possible to generalize to all hospitalized stroke patients in Sweden, this would result in a savings of 6,944,500 €/year. Further, when extrapolated, a 2-days shorter in-hospital stay would also mean a reduction in CO_2_ emissions of approximately 360,050 kg CO_2_/year. These hypothetical possible reductions in healthcare emissions would contribute to sustainable healthcare, reducing the contribution of healthcare activities to climate change and thereby benefit public health; as connections between climate change and health are a high priority, as discussed at the recent COP28 climate meeting in Dubai. Building on this, the Swedish National Council on Medical Ethics (SMER) has stated the importance of the healthcare system, with its mission to protect health, taking the lead in climate change mitigation and has urged prompt action to achieve net zero emissions [16].

Thirty-seven patients in the VESD group and 45 in the control group consumed primary care rehabilitation after discharge from the stroke unit, but there was no statistically significant difference between the groups. On average, the control group consumed one more visit compared with the VESD group. This is in line with studies showing no difference in proportion of independency or in the use of primary care rehabilitation between patients receiving VESD and patients receiving ordinary discharge [17, 18]. One might wonder why there is no statistical difference between the groups in how many needed primary care rehabilitation. One could imagine that those who received VESD, should have less need than the control group for continued rehabilitation via primary care rehabilitation after the VESD rehabilitation period. One explanation that there was no significant difference in the number of total primary care rehabilitation visits could be that the VESD team detected more hidden symptoms when they saw the patient in activity in their home environment and therefore referred them on to primary care rehabilitation after completing the VESD time. Another possible explanation is that the patients in the VESD group became aware of their symptoms and problems when they returned home and thanks to the close contact with the VESD team, they were able to ask for more rehabilitation. It may also be that those in the VESD group realized the value of rehabilitation and therefore wanted continued rehabilitation after they had been discharged from the VESD team. However, there was a significant difference in how many occupational therapy visits were consumed in the different groups, with the VESD group having more visits than the control group. This may also indicate that VESD patients became more aware of their rehabilitation needs, especially those related to hidden disabilities which may be easier to discover once home. Nevertheless, there was no significant difference in how the two groups estimated their health-related quality of life as measured by the Eq. 5D one year after discharge. This confirms that economic and environmental savings achieved from less healthcare consumption were not made at the expense of patients’ quality of life. This is consistent with some studies that have analysed quality of life after stroke depending on the form of rehabilitation [19], but there are also studies that have found differences between intervention and control groups in favour of the intervention group [20].

This study present some strengths. First this study is a randomised controlled study, were there was no statistical difference between the groups at baseline. To our knowledge, this is the first study to analyze the impact of rehabilitation on the environment estimated in kg carbon dioxide (CO_2_) which is a strength. Another strength is that we have data on economic cost due to rehabilitation the entire first year after stroke admission.

Some limitations of this study should be noted. One is that even though patients were randomized to VESD, the discharge was not expedited, which may be the reason why there was only a 2-day difference in the number of inpatients days between the groups. However, with today’s short care times, it can be difficult to discharge patients even earlier. A second limitation is that we have not had access to data on other outpatient care such as doctor’s visits, visits to the district nurse, etc. Therefore, we do not know to what extent the participants consumed other outpatient care, that could have influenced the total cost and environmental impact of healthcare consumption during the year. Another limitation is that there are no local or national data available on CO_2_ emissions from healthcare activities in the Sweden, and the estimates used in this study are based on those of the National Health Service in England, which may differ from the Swedish healthcare setting. Further, the calculated CO2 emissions from VESD visits may be overestimated, since they are based on the maximum distance of home visits according to the inclusion criteria. No information on transport mode for VESD visits was collected and therefore the true values of CO2 emissions from VESD visits are likely to be much smaller, since hospital policy regarding the use of sustainable staff transportation recommends hospital bicycles or public transportation as the first choice. Hence, the benefits of VESD for sustainable healthcare are likely to be larger than are estimated in this study. There was some limitations in the GOTVED study that should be mentioned; the set level for the inclusion assessments NIHSS and BI scores could have an impact on enrolment and that the screening was performed mainly on weekdays could have impact the inclusion. A second limitation was that the choice of inclusion window may have impacted the fact that we mainly captured patients with mild stroke due to our intention to capture patients with mild to moderate stroke. Lastly, the power calculation in the GOTVED study was not done on the outcome measures analyzed in this study, so the analyses in this study could be underpowered.

## Conclusion

Our study shows that VESD service can be a sustainable alternative to ordinary discharge routines for patients with disability after stroke because it can reduce the cost and environmental impact of healthcare consumption while maintaining patients’ quality of life. A policy of offering more patients VESD after stroke could reduce healthcare costs and environmental impact, contributing to the sustainability of healthcare.

## Data Availability

According to the Swedish regulations at https://etikprovning.se/for-forskare/ansvar/, for ethical and legal reasons the dataset presented in this article is not readily available. Request to access the dataset should be directed to the corresponding author.

## Abbreviations

AI: Artificial intelligence
CO_2_: Carbon dioxide
€: €o
GOTVED: Gothenburg Very Early Supported Discharge
IQR: Interquartile range
OR: ODDS ratio
SD: Standard deviation
SDU: Sustainable Development Unit
VESD: Very Early Supported Discharge
